# Moderate Exercise Stimulates PACAP-Mediated Neurogenesis in Rat Dentate Gyrus and Cerebellar Cortex

**DOI:** 10.3390/jfmk11010037

**Published:** 2026-01-15

**Authors:** Grazia Maugeri, Salvatore Di Bartolo, Nicoletta Palmeri, Agata Grazia D’Amico, Desiree Brancato, Concetta Federico, Velia D’Agata, Giuseppe Musumeci

**Affiliations:** 1 Department of Biomedical and Biotechnological Sciences, Section of Anatomy, Histology and Movement Science, School of Medicine, University of Catania, Via S. Sofia, 97, 95123 Catania, Italy; salvatore.dibartolo26@gmail.com (S.D.B.); nicoletta.palmeri@unict.it (N.P.); vdagata@unict.it (V.D.); g.musumeci@unict.it (G.M.); 2 Department of Drug Sciences, University of Catania, 95123 Catania, Italy; agata.damico@unict.it; 3 Department of Biological, Geological and Environmental Sciences, University of Catania, 95124 Catania, Italy; desiree.brancato@phd.unict.it (D.B.); federico@unict.it (C.F.)

**Keywords:** physical activity, neurogenesis, PACAP, DCX

## Abstract

**Background:** Moderate physical activity (PA) exerts powerful systemic and neuroprotective effects, reducing chronic disease risk and enhancing cognitive and psychological well-being. PA promotes brain plasticity by upregulating neurotrophic factors and stimulating neurogenesis. Given the established role of Pituitary Adenylate Cyclase-Activating Polypeptide (PACAP) in neuronal survival, differentiation, and anti-apoptotic signaling, we aimed to investigate whether moderate PA modulates the endogenous expression of PACAP and its specific receptor PAC1R in the DG and cerebellar cortex. **Methods:** To this end, twenty-four rats were distributed into sedentary or exercise groups. Immunohistochemical and Western blot analyses were performed to assess PACAP and PAC1R expression. Co-expression with doublecortin (DCX), a marker of immature neurons, was evaluated to explore the direct relationship between PACAP signaling and neurogenesis. **Results:** Our results showed that moderate PA induced a significant up-regulation of PACAP and PAC1R in both the DG and cerebellar cortex compared to sedentary controls. Moreover, high co-expression of PACAP and DCX was detected in these regions, suggesting an involvement of PACAP in exercise-induced neurogenic processes. **Conclusions:** These findings demonstrate that moderate physical activity is associated with enhanced PACAP/PAC1R signaling and DCX expression in neurogenic regions, warranting further investigation into its specific contribution to exercise-induced brain plasticity.

## 1. Introduction

Moderate physical activity (PA) is a powerful rejuvenator for the body, as established and supported by numerous studies in recent years. Moderate PA offers a wide range of systemic benefits, including the reduction in chronic conditions and the prevention of cardiovascular diseases, inflammation, obesity, hypertension, and type 2 diabetes [1,2]. Recent research also suggests that an enriched environment (EE), which combines social stimulation with physical activity, can lead to both structural and functional changes in the brain. In particular, moderate PA plays a key role in enhancing brain plasticity and supporting psychological well-being [3,4]. Furthermore, exercise aids recovery from brain injuries such as stroke by enhancing the expression of neurotrophic factors like Brain-Derived Neurotrophic Factor (BDNF), promoting growth, survival, the differentiation of neurons, angiogenesis, and stimulating anti-apoptotic pathways [1,3,4]. Additionally, exercise exhibits neuroprotective effects against neurodegenerative diseases, including Parkinson’s disease (PD), Alzheimer’s disease (AD), Amyotrophic Lateral Sclerosis, and Huntington’s disease [5,6,7,8]. Aerobic exercise was shown to increase hippocampal volume and perfusion, which correlates with improvements in cognitive function, learning, and visuospatial memory, functions primarily regulated by the hippocampus [1,3,9,10]. Studies performed in rodent models have demonstrated that both forced running on a treadmill and voluntary wheel running increase the number of new neurons in the hippocampus and improve spatial learning [11,12,13]. These findings highlight the important role of physical activity in enhancing Long-Term Potentiation (LTP) and adult hippocampal neurogenesis. Such effects help mitigate age-related hippocampal shrinkage, which is associated with an increased risk of dementia, thus positioning moderate physical activity as a key factor in “brain rejuvenation” [1,5,9,14].

It is well established that neurogenesis, albeit to a lesser extent, also occurs in the adult brain [4,12,15]. Specifically, new neurons are generated in the subgranular zone (SGZ) of the dentate gyrus (DG) within the hippocampus, and in the subventricular zone (SVZ) of the lateral ventricles. These two regions are recognized as the primary neurogenic sites responsible for adult hippocampal neurogenesis [4,5,10,12,16,17]. Interestingly, moderate PA was shown to promote the neurogenesis process in the aforementioned brain regions. [4,13,15,18]. Although adult neurogenesis is primarily limited to certain brain regions, recent studies have highlighted neurogenic activity in other brain areas, such as the cerebellum [17,19], which plays a key role in motor coordination, cognitive processing, and sensory discrimination [17,20,21]. Focusing on the effect of physical activity on the cerebellum, increased mitochondrial function, cerebellar plasticity, and inhibition of apoptosis in Purkinje cells have been observed [12,17,22,23].

Pituitary Adenylate Cyclase Activating Polypeptide (PACAP) is a pleiotropic neuropeptide [24,25] that exerts neurotrophic and neuroprotective functions. It was originally isolated from the ovine hypothalamus, encoded by the highly conserved gene *ADCYAP1,* which belongs to the vasoactive intestinal polypeptide (VIP) secretin-glucagon peptide superfamily [26]. PACAP exists in two isoforms, PACAP 38 and PACAP 27, and it is expressed not only in the central nervous system (CNS) but also in peripheral organs [25,27,28,29]. PACAP interacts with three different G-protein-coupled receptors: PAC1R, which is the selective one, and VPAC1 and VPAC2, shared with VIP. These receptors predominantly couple to the G_s_ protein to activate the adenylate cyclase (AC) to produce cAMP. The elevated cAMP stimulates PKA (protein kinase A), which in turn activates the MAPK signaling pathway. This cascade promotes cell proliferation and exerts an anti-apoptotic effect [25,29,30,31,32,33,34]. The protective role of PACAP has been shown in various models of brain injuries, including cerebral ischemia, Parkinson’s disease, Alzheimer’s disease, UV-A-induced retinal damage, and diabetic retinopathy [25,27,35,36,37,38]. PACAP is expressed in the CNS from development to adulthood, carrying out its neuroprotective functions [32]. The neuropeptide and its receptors are expressed in the hippocampal DG of mature rat brains [24,31] and in the developing cerebellum of adult rats, in particular in the Purkinje cells [30,33,39], suggesting that PACAP may modulate the histogenesis of the cerebellar cortex [29].

The therapeutic potential of exogenous PACAP has been widely documented; however, its clinical application is often hampered by rapid systemic degradation and poor permeability across the blood–brain barrier. Consequently, there is a growing interest in identifying non-invasive strategies capable of endogenously modulating the PACAP/PAC1R axis. Moderate PA is a well-known inducer of brain plasticity and neurogenesis, yet a significant research gap exists regarding whether these systemic benefits are mediated by an upregulation of the endogenous PACAP system in neurogenic niches. Unlike pharmacological interventions, PA-induced modulation reflects a physiological recruitment of neuroprotective pathways. By investigating the expression of PACAP, its preferring receptor PAC1R, and doublecortin (DCX) in the DG and in the cerebellum of sedentary rats and rats performing moderate training, this study aims to establish a functional link between lifestyle-induced endogenous signaling and the stimulation of immature neuronal populations in the neurogenic regions. Our findings indicate that moderate exercise significantly increases the expression of both the peptide and its receptor within the investigated rat brain regions. Moreover, we found an interesting co-expression of PACAP and DCX, a proliferative marker of the immature neurons, both in the DG and in the cerebellum, suggesting that the role of physical activity in promoting neurogenesis could be mediated in part by PACAP expression.

## 2. Materials and Methods

### 2.1. Ethical Approval

All procedures adhered to the guidelines outlined by the Institutional Animal Care and Use Committee (I.A.C.U.C.) of the University of Catania (Protocol No. 2112015-PR dated 14 January 2015, approved by the Italian Ministry of Health). The experiments were conducted in compliance with the European Community Council Directive (86/609/EEC) and the Italian Animal Protection Law (Law No. 116/1992). The entire experimentation was performed at the “Center for Advanced Preclinical In Vivo Research (CAPIR)”.

### 2.2. Animals

Twenty-four healthy male adult Wistar Outbred Rats (3-month-old, 300 ± 20 g) were purchased from the Charles River Laboratories (Milan, Italy). All rats were housed in an environment of a 12 h light/dark cycle, a temperature of 20  ±  3 °C, 55% humidity, and ad libitum standard diet and water. The rats were humanely sacrificed by a lethal intravenous injection of an anesthetic overdose, and brain samples were explanted and fixed in paraformaldehyde for the immunohistochemical analysis and Western blot analysis, as previously described [1]. Regarding the sample size determination, an initial power analysis was performed using G*Power 3.1 (version 3.1.9.2, Heinrich-Heine-Universität Düsseldorf, Düsseldorf, Germany). To achieve a power of 0.80 with a predicted large effect size (d = 0.8) and α = 0.05, the analysis initially suggested a sample size of n = 26 rats per group. However, in strict adherence to the 3Rs principles (Replacement, Reduction, and Refinement) and according to the guidelines of our Institutional Animal Care and Use Committee (CAPIR), the number of animals was reduced to n = 12 per group. This decision was justified by the ‘Resource Equation’ method, which yielded an *E* value of 22, providing an adequate degree of freedom for the error term. Furthermore, this sample size was successfully utilized in our previous similar study [1]. A post hoc power verification based on our primary outcome (means: 0.17 ± 0.12 vs. 0.47 ± 0.12) revealed an observed effect size of d = 2.50, resulting in an actual statistical power of >0.99. This confirms the study was robustly powered to support its conclusions while ethically minimizing animal use.

### 2.3. Experimental Design

**Figure 1:**
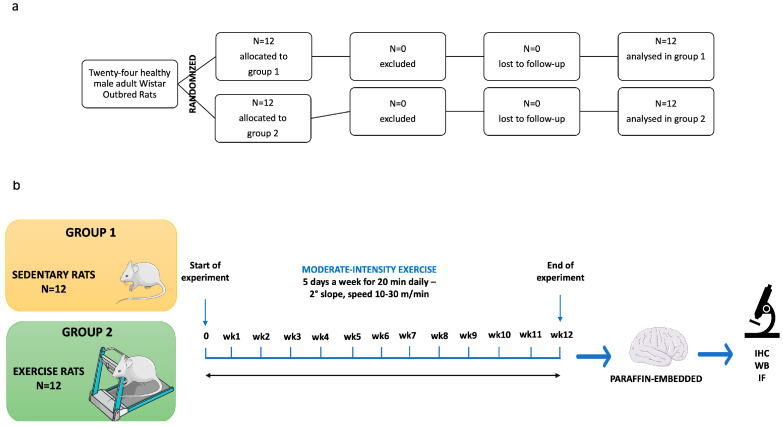
(**a**) The study consort flow diagram. (**b**) The diagrammatic representation of the processes involved in our research. IHC: immunohistochemistry analysis; WB: Western blot analysis; IF: immunofluorescence analysis.

The twenty-four rats, identified only by unique ID numbers, were randomly assigned to experimental groups (n = 12 per group) using a computer-generated random sequence. Animals were allocated to either Group 1 (sedentary control) or Group 2 (12-week moderate physical exercise on a treadmill, as per Di Rosa et al. [40]). To ensure allocation concealment, the sequence was placed in sequentially numbered, opaque, sealed envelopes. A researcher generated the sequence, while another researcher, who was blinded to the group assignments, enrolled the animals and opened the envelopes only after baseline measurements were completed. To maintain blinding during the study, cages were labeled with neutral codes (Group A and Group B) by a technician not involved in the outcome assessment. All outcome assessors, laboratory analysts, and the statistician remained blinded to the identity of these codes. The key linking the neutral labels to the actual treatment groups was kept in a locked file and was only unblinded after the formal statistical analysis was completed. In the training protocol, the rats trained 5 days a week, 20 min per day on a treadmill (2Biological instrument, Varese, Italy), with a slope set to 2°, and the speed was gradually raised from 10 to 30 m/min in order to stimulate moderate intensity training (Figure 1). Animals unable to perform exercise would be excluded and replaced. In our case, no animals were excluded from the study, and the results included all variables analyzed.

### 2.4. Immunohistochemistry (IHC) Analysis

The immunohistochemical analysis was used to evaluate the expression and distribution of PACAP and DCX in the DG and cerebellum of sedentary and trained rats, as previously described by Maugeri et al. [1].

The immunoreactivity was assessed using a 3,3′-diaminobenzidine solution (DAB substrate Kit; SK-4100, Vector Laboratories, Burlingame, CA, USA). After that, the samples were colored with hematoxylin as a counterstain. The sample observation was performed using an Axioplan Zeiss light microscope (Carl Zeiss Microscopy GmbH, Jena, Germany), and the digital micrographs were captured with a digital camera (AxioCam MRc5, Carl Zeiss) via AxioVision Release 4.8.2—SP2 Software (version 4.8.2, Carl Zeiss Microscopy GmbH, Jena, Germany).

### 2.5. FFPE Tissue Samples

The formalin-fixed and paraffin-embedded brain tissue samples were cut using the microtome into 16 µm thick tissue slices from the hippocampus and cerebella of group 1 (sedentary) and group 2 (trained) and collected in tubes. The tissue sections were deparaffinized and rehydrated using xylene and a graded ethanol series (100%, 96%, and 70%) to extract the proteins for the Western blot analysis. Protein extraction was conducted using Qproteome FFPE Tissue Extraction Buffer (Qiagen, Hilden, Germany). The pellet of each tube was added with an extraction buffer consisting of β-mercaptoethanol and incubated at 100 °C for 20 min and at 80 °C for 2 h. Subsequently, the tubes were centrifuged for 15 min at 14,000 rpm. Then, the supernatants were collected in tubes, and protein concentrations were quantified by using the Quant-iT Protein Assay Kit (Invitrogen, Carlsbad, CA, USA).

### 2.6. Western Blot Analysis

About 15 µg of proteins were diluted in 2× Laemmli buffer (Invitrogen, Carlsbad, CA, USA) and heated at 70 °C for 10 min, as previously described [41]. Proteins were separated on a Bio-Rad Criterion XT Bis-Tris 4–15% and then electro-transferred to a nitrocellulose membrane (Bio-Rad Inc., Hercules, CA, USA). The Odyssey Blocking buffer (Li-Cor Biosciences, Lincoln, NE, USA) was used to block the blots for 1 h. The membranes were incubated overnight at 4 °C with specific primary antibodies: rabbit anti-DCX (Doublecortin polyclonal antibody, REF. 48-1200, Invitrogen), mouse anti-PACAP (Santa Cruz, CA, USA, SC-166180), mouse anti-PAC-1R (Santa Cruz, SC-100315), and mouse anti-β-actin (Santa Cruz, SC-47778). The secondary antibodies, goat anti-rabbit IRDye 800CW (926-32211; Li-Cor Biosciences) and goat anti-mouse IRDye 680CW (926-68020D, Li-Cor Biosciences), were used at 1:20,000 and 1:30,000, respectively. Blots were scanned with an Odyssey Infrared Imaging System (Odyssey, Li-Cor Biosciences, Lincoln, NE, USA). Original immunoblots are reported in Supplementary Figures S1 and S2. Densitometric analyses of Western blot signals were evaluated using the ImageJ software (version 1.54g, NIH, Bethesda, MD, USA; available at http://rsb.info.nih.gov/ij/index.html (accessed on 7 November 2025)). The Region of Interest for each band was defined using a fixed-size rectangular frame that encompassed the largest band in the series to ensure consistency across all lanes. The values were normalized using β-actin as a loading control.

### 2.7. Immunofluorescence Analysis

To determine the cellular distribution and co-localization of PACAP and DCX in the DG and cerebellum of sedentary and trained rats, immunofluorescence analysis (IF) was performed as previously described [42]. The sections were incubated overnight at 4 °C with specific primary antibodies: rabbit anti-DCX (REF. 48-1200), mouse anti-PACAP (SC-166180), mouse anti-PAC-1R (SC-100315), and mouse anti-β-actin (SC-47778). Signals were revealed with Alexa Fluor 488 goat anti-rabbit and Alexa Fluor 594 goat anti-mouse, for 1.5 h at room temperature (shielded from light). DNA was counter-stained with 4,6-diamidino-2-phenylindole (DAPI; cat. no 940110; Vector Laboratories, Burlingame, CA, USA). Immunolocalization was analyzed by confocal laser scanning microscopy (Zeiss LSM700, Oberkochen, Germany).

### 2.8. Statistical Analysis

Data were analyzed using GraphPad Prism 9 (GraphPad Software, La Jolla, CA, USA). All values are presented as means ± SEM. Distribution normality was assessed using the Shapiro–Wilk test. Statistical significance was assessed via an unpaired two-tailed Student’s *t* test. The level of significance for all statistical tests was set at *p* ≤ 0.05.

## 3. Results

### 3.1. Effect of Moderate Training on PACAP and PAC1R Expression in the Rat DG and Cerebellum

**Figure 2:**
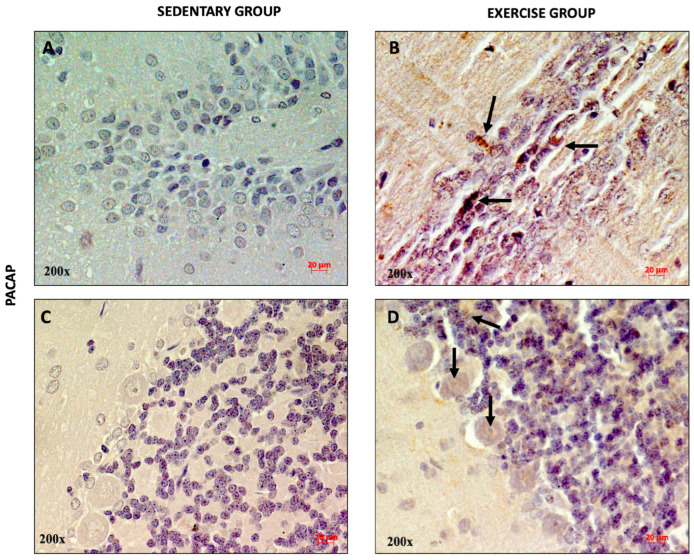
Representative immunohistochemical staining of PACAP in DG (n = 12) (**A**,**B**) and cerebellum (n = 12) (**C**,**D**) of sedentary rats and rats performing moderate training. Digital micrographs are representative results of fields taken in randomly selected slides and obtained using the Zeiss Axioplan light microscope, fitted with a digital camera. The black arrows indicate PACAP-immunopositive cells. Scale bar: 20 μm. Magnification: 200×.

To qualitatively assess the distribution of PACAP and PAC1R in the DG and cerebellar cortex of rats performing moderate training, immunohistochemical analysis was performed. As shown in Figure 2A, a weak immunopositivity for PACAP was found in the dentate gyrus and in some cells of the cerebellar granule layer of sedentary rats (Figure 2C). Interestingly, the expression of the peptide was higher in rats performing moderate training. In fact, several PACAP-immunopositive neurons (indicated with black arrows) were detected in the DG (Figure 2B) and in the soma of Purkinje cells, as well as in the granule cells (Figure 2D).

**Figure 3:**
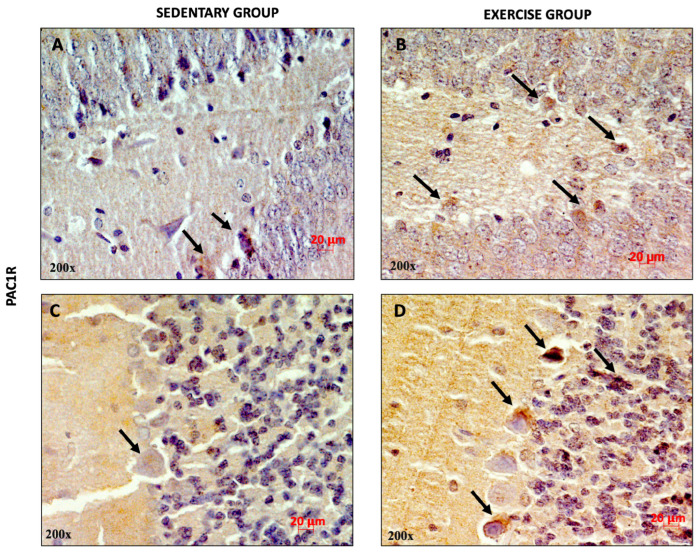
Representative immunohistochemical staining of PAC1R in DG (n = 12) (**A**,**B**) and cerebellum (n = 12) (**C**,**D**) of sedentary rats and rats performing moderate training. Digital micrographs are representative results of fields taken in randomly selected slides and obtained using the Zeiss Axioplan light microscope, fitted with a digital camera. The black arrows refer to the PAC1R immunopositive cells. Scale bar: 20 μm. Magnification: 200×.

Similarly, high PAC1R expression was observed in the DG and in the cerebellar cortex of training rats (Figure 3B,D) compared to sedentary rats, suggesting that moderate physical activity increased the expression levels of the peptide and its receptor.

### 3.2. Moderate Training Promotes Adult Neurogenesis

**Figure 4:**
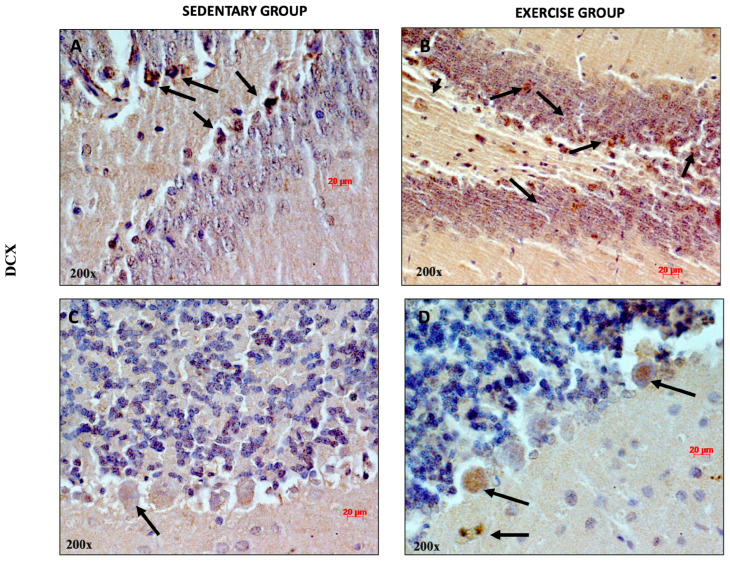
Representative immunohistochemical staining of DCX in DG (n = 12) (**A**,**B**) and cerebellum (n = 12) (**C**,**D**) of sedentary rats and rats performing moderate training. Digital micrographs are representative results of fields taken in randomly selected slides and obtained using the Zeiss Axioplan light microscope, fitted with a digital camera. The black arrows refer to the DCX immunopositive cells. Scale bar: 20 μm. Magnification 200×.

Exercise exerts a profound effect on the structure and function of the brain. Several studies have shown that exercise promotes neuroplasticity, increases dendritic density, and promotes neurogenesis [43,44]. To determine whether moderate training induces adult neurogenesis, we analyzed the expression and distribution of DCX, considering that this protein is associated with neuronal differentiation and synaptogenesis [45]. As shown in Figure 4A, DCX-positive cells were detected both in the DG of sedentary and training rats (some of them indicated with black arrows). Faint DCX immune-positive staining was observed in the cerebellar cortex of sedentary rats. This result is in accord with previous studies showing that DCX is more greatly expressed both in cerebellar Purkinje cells and granule cells in rat pups aged two and seven days [46]. It is noteworthy that higher immunopositivity for DCX was detected in the soma of Purkinje cells and in granule cells of training rats, suggesting that physical activity promotes adult neurogenesis in the cerebellum. This evidence is corroborated by a previous study showing that the cells of the adult cerebellum expanded after adequate physical exercise in mice [47].

**Figure 5:**
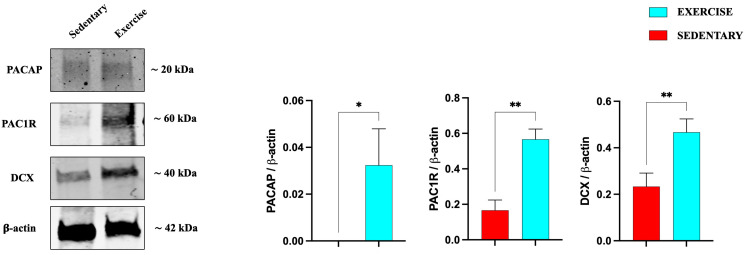
Representative immunoblots of PACAP, PAC1R, and DCX expression in hippocampus of sedentary (n = 12) and training (n = 12) rats. The bar graph shows quantitative analysis of signals obtained by immunoblots resulting from three independent experiments. Relative band densities were quantified using ImageJ software. Protein levels are expressed as arbitrary units obtained following normalization to β-actin, which was used as loading control. Data represent means ± SEM. * *p* < 0.05 and ** *p* < 0.01 vs. sedentary, as determined by unpaired two-tailed Student *t*-test.

We also investigated the expression of PACAP, PAC1R, and DCX in the serial brain sections (coming from the same paraffin-embedded samples used for IHC analysis) of sedentary and training rats through Western blot analysis. As shown in Figure 5, the expression levels of the peptide, its receptor, and DCX are significantly increased in the DG of rats performing moderate training compared to the sedentary group.

**Figure 6:**
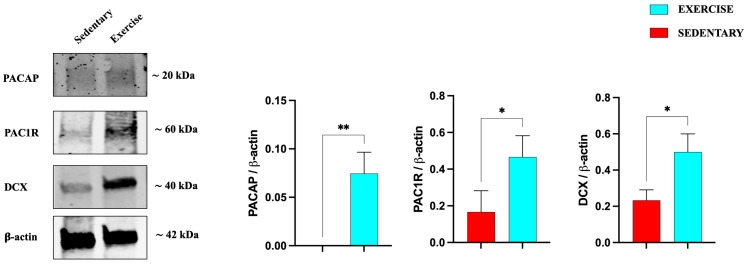
Representative immunoblots of PACAP, PAC1R, and DCX expression in cerebellum of sedentary (n = 12) and training (n = 12) rats. The bar graph shows quantitative analysis of signals obtained by immunoblots resulting from three independent experiments. Relative band densities were quantified using ImageJ software. Protein levels are expressed as arbitrary units obtained following normalization to β-actin, which was used as loading control. Data represent means ± SEM. * *p* < 0.05 and ** *p* < 0.01 vs. sedentary, as determined by unpaired two-tailed Student *t*-test.

Similarly to the hippocampus results, also in the cerebellum (Figure 6), the expression levels of PACAP, its receptor, and DCX are significantly increased in training rats, confirming the positive role of moderate exercise in inducing the expression of this neurotrophic factor, as well as promoting adult neurogenesis.

### 3.3. Moderate Training Promotes the Co-Localization of PACAP and DCX in Rat DG and Cerebellum

**Figure 7:**
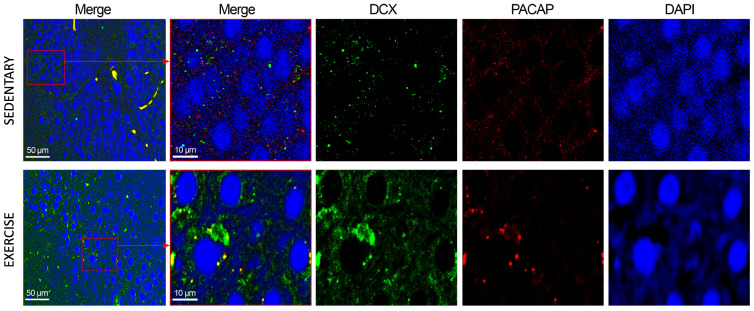
Co-localization of DCX and PACAP in DG of sedentary (n = 12) and training rats (n = 12). Representative photomicrographs show MERGE (yellow), DCX expression (green), PACAP (red), and DAPI (blue). Photomicrographs are representative results of fields taken randomly from each slide. Scale bar: 50 µm and 10 µm.

To assess the subcellular co-localization of PACAP and DCX, IF staining was performed. As depicted in Figure 7, in the DG of sedentary animals, PACAP and DCX exhibited low-level co-expression, primarily localized to the perinuclear compartment and cytoplasm. In animals subjected to moderate physical training, DCX immunoreactivity was markedly upregulated, corroborating data obtained from immunohistochemistry and Western blot analyses. Furthermore, certain cells demonstrated robust PACAP immunostaining that co-localized with DCX, suggesting potential functional interplay in neurogenic regions under exercise-induced conditions.

**Figure 8:**
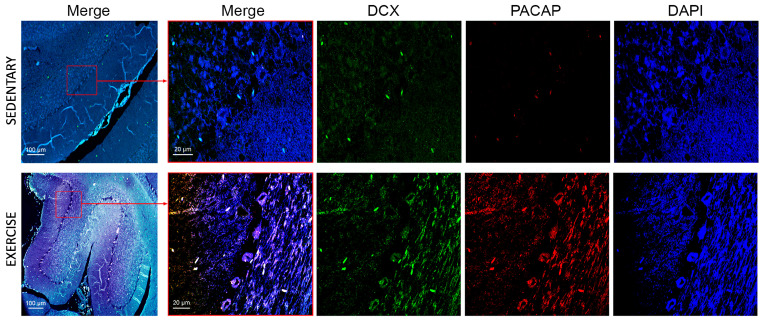
Co-localization of DCX and PACAP in cerebellum of sedentary (n = 12) and training rats (n = 12). Representative photomicrographs show MERGE (yellow), DCX expression (green), PACAP (red), and DAPI (blue). Photomicrographs are representative results of fields taken randomly from each slide. Scale bar: 100 µm and 20 µm.

In the cerebellum of sedentary rats, DCX expression levels were markedly low. Conversely, in the cerebellum of active rats, DCX expression was significantly upregulated, predominantly in Purkinje cells and the granule cell layer (Figure 8), thereby confirming the stimulatory effect of moderate physical activity on DCX expression. In contrast, only a limited number of immunopositive signals for PACAP were observed, supporting the negligible expression of this peptide in the cerebellum of sedentary rats. Notably, Nielsen et al. [39] previously demonstrated that PACAP-38 concentrations are elevated postnatally but decline progressively during the first few weeks in the adult rat cerebellar cortex. In the cerebellum of training rats, PACAP was markedly expressed in all three layers of the cerebellum and co-localized with DCX, confirming the role of physical activity in promoting expression and co-localization of PACAP and DCX.

## 4. Discussion

PA is widely recognized as an effective non-pharmacological strategy for both preventing and managing various pathological conditions, as well as for promoting overall health [48,49,50,51,52,53,54]. The positive impact of PA across several neurodegenerative disorders strengthens the hypothesis that exercise exerts a neuroprotective effect. Engaging in regular exercise upregulates genes associated with enzymatic antioxidant defenses, enhances cognitive performance and memory, and may slow disease progression or help individuals preserve their ability to carry out daily activities. These benefits are likely mediated through multiple cellular and molecular pathways that operate synergistically [55,56].

Extensive research has highlighted the role of PA in activating quiescent stem cells, restoring proliferation capacity, and inducing neurogenic niche remodeling [57]. In fact, in rodent studies, moderate PA significantly increased the amount of DCX + cells in the DG [15,58,59]. DCX, a 40 kDa phosphoprotein encoded by the *DCX* gene, is a nervous system-specific microtubule-associated protein expressed in migrating neurons of the central and peripheral nervous system during embryonic and postnatal development, commonly used as a reliable marker of immature neurons and neurogenesis [60,61]. Our results clearly demonstrated that rats performing moderate PA express higher levels of DCX in the DG compared to the sedentary group, confirming the direct role played by aerobic PA in neurogenesis. Physical exercise exerts neurogenic effects not only in DG, but also in the adult cerebellum, as reported by the increased expression of Sox2 in the Purkinje cell layer of transgenic mice exposed to PA and an enriched environment [47]. Accordingly, our data showed higher expression of DCX in the soma of Purkinje cells and in granule cells of training rats compared to sedentary animals. The identification of increased DCX expression in the adult cerebellum suggests that this region possesses a higher degree of structural flexibility. From a clinical perspective, these findings imply that the adaptive response of the cerebellum could be pharmacologically or physically—for instance, through moderate PA—modulated to enhance recovery following localized injury or neurodegeneration. Therefore, rather than viewing the adult cerebellum as a static circuit, these DCX-positive populations suggest a dynamic environment where synaptic or cellular remodeling remains possible.

PACAP is a small polypeptide with considerable neuroprotective properties in both in vitro and in vivo models [62,63]. Notably, PACAP expression increases in response to brain ischemia [64,65,66] and in cortical regions following traumatic brain injury [67], suggesting that it may function as an endogenous mediator that facilitates neural repair under conditions of neurodegenerative stress. Beyond its neuroprotective effects, PACAP and its receptor PAC1 have been reported to influence neural progenitor cells in both embryonic and adult nervous systems [24,63,68,69]. In the immature cerebellum, PACAP and PAC1R are intensely expressed. This suggests that PACAP may modulate the histogenesis of the cerebellum cortex, promoting cell survival due to the inhibition of caspases [29]. Another study analyzed how PACAP could stimulate adult neural stem cell proliferation in vitro [24]. In this particular case, PACAP was infused into the lateral ventricle of adult mice, and the incorporation of BrdU highlighted an increased number of BrdU-labeled cells in the DG of the hippocampus and SVZ. On the other hand, Ago et al. [31] examined the effect of an enriched environment on wild-type and PACAP^−/−^ mice. The results showed an increase in the survival of newly divided cells in wild-type mice, induced by an enriched environment, whereas in the PACAP^−/−^ mice, enriched environment-induced survival was reduced. The proliferation of newly divided cells in the SGZ did not differ between wild-type and PACAP^−/−^ mice subjected to enriched environment rearing, whereas Mercer et al. [24] showed how exogenous PACAP infusion stimulates cell proliferation in the hippocampi of adult mice.

Considering the effect of exercise in stimulating neurogenesis and the active role of PACAP in this process, the aim of this study was to evaluate whether moderate PA alters the endogenous expression of PACAP and PAC1R in the DG and cerebellum of adult rats. The effect of exercise on PACAP/PAC1R expression was previously demonstrated in mice with Alzheimer’s disease (AD) [70]. In this model, moderate PA protected the kidneys from Aβ accumulation and rescued PACAP receptor expression as well as PACAP-mediated signaling, indicating that the neuroprotective action of physical activity in AD might be, at least partly, mediated by PACAP.

Our study demonstrated a significant up-regulation of endogenous PACAP and its specific receptor PAC1R in brain regions associated with neurogenesis and motor coordination. In particular, in the hippocampus of rats subjected to moderate PA, we observed a marked increase in the co-expression of PACAP and DCX within the dentate gyrus. This pattern suggests that PACAP signaling is closely associated with neuronal differentiation processes occurring during exercise-induced neurogenesis. Considering the neurotrophic and anti-apoptotic roles played by PACAP, its upregulation alongside DCX supports the idea that the neuropeptide contributes to the survival and maturation of newly generated neurons under conditions of moderate physical stimulation. Our data clearly showed high co-expression levels of PACAP and DCX in the cerebellar cortex, and, in particular, in the Purkinje cells and cerebellar granule cells of rats performing moderate physical activity. These results suggested that exercise induces PACAP signaling, which in turn could promote DCX expression as part of cytoskeletal reorganization or differentiation processes. Moreover, moderate PA stimulates the PACAP-mediated modulation of neurogenic or neuroprotective pathways. Accordingly, cerebellar granule cells, cultured under serum-free medium, die within 48 h of culture, whereas treatment with subnanomolar concentrations of PACAP promotes cell survival and induces the appearance of a dense network of long neurites, confirming an enhanced neuronal plasticity [30].

Despite these findings, several limitations should be considered. Firstly, our experiments were conducted exclusively in adult male rats. Future studies should include females to determine whether there are sex-dependent differences in the expression of this neurotrophic factor. In addition, it would be valuable to examine whether the absence of PACAP alters the effects of moderate physical activity on neurogenesis by comparing wild-type and PACAP-knockout rats. Such studies could also employ a broader panel of neurogenesis markers and include animals at different developmental stages to provide a more comprehensive understanding of PACAP’s role in exercise-induced neurogenesis. Moreover, we used only DCX as a marker for neurogenesis; therefore, future studies with mature neuronal markers like NeuN are needed. Furthermore, longitudinal behavioral assays or electrophysiological recordings will be essential to determine if this increase in immature neurons translates into functional integration and improved cognitive outcomes. In conclusion, moderate exercise is associated with upregulated PACAP/PAC1R and DCX expression in the DG and cerebellar cortex, suggesting a possible role in exercise-related neuroplasticity that requires further mechanistic validation.

## Data Availability

The original contributions presented in this study are included in the article/Supplementary Materials. Further inquiries can be directed to the corresponding author.

## Supplementary Materials

The following supporting information can be downloaded at https://www.mdpi.com/article/10.3390/jfmk11010037/s1.

## Abbreviations

The following abbreviations are used in this manuscript:

AC	Adenylate cyclase
AD	Alzheimer’s disease
BDNF	Brain-derived neurotrophic factor
cAMP	Cyclic adenosine monophosphate
CNS	Central nervous system
DAB	Diaminobenzidine solution
DCX	Doublecortin
DG	Dentate gyrus
IF	Immunofluorescence
LTP	Long-term potentiation
MAPK	mitogen-activated protein kinases
PA	Moderate physical activity
PAC1R	Pituitary adenylate cyclase-activating polypeptide type I receptor
PACAP	Pituitary adenylate cyclase-activating polypeptide
PD	Parkinson’s disease
PKA	Protein kinase A
SGZ	Subgranular zone
SVZ	Subventricular zone
UV-A	Ultraviolet radiation-A
VIP	Vasoactive intestinal polypeptide
VPAC1	Vasoactive intestinal peptide receptor 1
VPAC2	Vasoactive intestinal peptide receptor 2

## References

[1] Maugeri G., D’amico A.G., Federico C., Saccone S., D’agata V., Musumeci G. (2024). Moderate Physical Activity Increases the Expression of ADNP in Rat Brain. Int. J. Mol. Sci..

[2] Makizako H., Liu-Ambrose T., Shimada H., Doi T., Park H., Tsutsumimoto K., Uemura K., Suzuki T. (2015). Moderate-intensity physical activity, hippocampal volume, and memory in older adults with mild cognitive impairment. J. Gerontol. A Biol. Sci. Med. Sci..

[3] Ben-Zeev T., Shoenfeld Y., Hoffman J.R. (2022). The Effect of Exercise on Neurogenesis in the Brain. Isr. Med. Assoc. J..

[4] van Praag H., Kempermann G., Gage F.H. (1999). Running increases cell proliferation and neurogenesis in the adult mouse dentate gyrus. Nat. Neurosci..

[5] Kim J.W., Nam S.M., Yoo D.Y., Jung H.Y., Kim I.Y., Hwang I.K., Seong J.K., Yoon Y.S. (2017). Comparison of Adult Hippocampal Neurogenesis and Susceptibility to Treadmill Exercise in Nine Mouse Strains. Neural Plast..

[6] Maugeri G., D’Agata V. (2020). Effects of Physical Activity on Amyotrophic Lateral Sclerosis. J. Funct. Morphol. Kinesiol..

[7] Sujkowski A., Hong L., Wessells R., Todi S.V. (2022). The protective role of exercise against age-related neurodegeneration. Ageing Res. Rev..

[8] Paillard T., Rolland Y., de Souto Barreto P. (2015). Protective Effects of Physical Exercise in Alzheimer’s Disease and Parkinson’s Disease: A Narrative Review. J. Clin. Neurol..

[9] Erickson K.I., Voss M.W., Prakash R.S., Basak C., Szabo A., Chaddock L., Kim J.S., Heo S., Alves H., White S.M. (2011). Exercise training increases size of hippocampus and improves memory. Proc. Natl. Acad. Sci. USA.

[10] Lee M.C., Inoue K., Okamoto M., Liu Y.F., Matsui T., Yook J.S., Soya H. (2013). Voluntary resistance running induces increased hippocampal neurogenesis in rats comparable to load-free running. Neurosci. Lett..

[11] van Praag H., Shubert T., Zhao C., Gage F.H. (2005). Exercise enhances learning and hippocampal neurogenesis in aged mice. J. Neurosci..

[12] Thomas A.G., Dennis A., Bandettini P.A., Johansen-Berg H. (2012). The effects of aerobic activity on brain structure. Front. Psychol..

[13] Gomes da Silva S., Arida R.M. (2015). Physical activity and brain development. Expert. Rev. Neurother..

[14] Vecchio L.M., Meng Y., Xhima K., Lipsman N., Hamani C., Aubert I. (2018). The Neuroprotective Effects of Exercise: Maintaining a Healthy Brain Throughout Aging. Brain Plast..

[15] Uda M., Ishido M., Kami K., Masuhara M. (2006). Effects of chronic treadmill running on neurogenesis in the dentate gyrus of the hippocampus of adult rat. Brain Res..

[16] Pereira A.C., Huddleston D.E., Brickman A.M., Sosunov A.A., Hen R., McKhann G.M., Sloan R., Gage F.H., Brown T.R., Small S.A. (2007). An in vivo correlate of exercise-induced neurogenesis in the adult dentate gyrus. Proc. Natl. Acad. Sci. USA.

[17] Leal-Galicia P., Chávez-Hernández M.E., Mata F., Mata-Luévanos J., Rodríguez-Serrano L.M., Tapia-De-Jesús A., Buenrostro-Jáuregui M.H. (2021). Adult Neurogenesis: A Story Ranging from Controversial New Neurogenic Areas and Human Adult Neurogenesis to Molecular Regulation. Int. J. Mol. Sci..

[18] Zhao X., van Praag H. (2020). Steps towards standardized quantification of adult neurogenesis. Nat. Commun..

[19] Carletti B., Rossi F. (2008). Neurogenesis in the cerebellum. Neuroscientist.

[20] Andreotti J.P., Prazeres P.H., Magno L.A., Romano-Silva M.A., Mintz A., Birbrair A. (2018). Neurogenesis in the postnatal cerebellum after injury. Int. J. Dev. Neurosci..

[21] Wizeman J.W., Guo Q., Wilion E.M., Li J.Y. (2019). Specification of diverse cell types during early neurogenesis of the mouse cerebellum. Elife.

[22] Rauf S., Soesatyo M.H., Agustiningsih D., Partadiredja G. (2020). Moderate intensity intermittent exercise upregulates neurotrophic and neuroprotective genes expression and inhibits Purkinje cell loss in the cerebellum of ovariectomized rats. Behav. Brain Res..

[23] Mercurio S., Serra L., Nicolis S.K. (2019). More than just Stem Cells: Functional Roles of the Transcription Factor Sox2 in Differentiated Glia and Neurons. Int. J. Mol. Sci..

[24] Mercer A., Rönnholm H., Holmberg J., Lundh H., Heidrich J., Zachrisson O., Ossoinak A., Frisén J., Patrone C. (2004). PACAP promotes neural stem cell proliferation in adult mouse brain. J. Neurosci. Res..

[25] D’amico A.G., Maugeri G., Musumeci G., Reglodi D., D’agata V. (2021). PACAP and NAP: Effect of Two Functionally Related Peptides in Diabetic Retinopathy. J. Mol. Neurosci..

[26] Vaudry D., Gonzalez B.J., Basille M., Yon L., Fournier A., Vaudry H. (2000). Pituitary adenylate cyclase-activating polypeptide and its receptors: From structure to functions. Pharmacol. Rev..

[27] Matsumoto M., Nakamachi T., Watanabe J., Sugiyama K., Ohtaki H., Murai N., Sasaki S., Xu Z., Hashimoto H., Seki T. (2016). Pituitary Adenylate Cyclase-Activating Polypeptide (PACAP) Is Involved in Adult Mouse Hippocampal Neurogenesis After Stroke. J. Mol. Neurosci..

[28] Basille M., Gonzalez B.J., Desrues L., Demas M., Fournier A., Vaudry H. (1995). Pituitary adenylate cyclase-activating polypeptide (PACAP) stimulates adenylyl cyclase and phospholipase C activity in rat cerebellar neuroblasts. J. Neurochem..

[29] Falluel-Morel A., Chafai M., Vaudry D., Basille M., Cazillis M., Aubert N., Louiset E., DeJouffrey S., Le Bigot J.F., Fournier A. (2007). The neuropeptide pituitary adenylate cyclase-activating polypeptide exerts anti-apoptotic and differentiating effects during neurogenesis: Focus on cerebellar granule neurones and embryonic stem cells. J. Neuroendocrinol..

[30] Botia B., Basille M., Allais A., Raoult E., Falluel-Morel A., Galas L., Jolivel V., Wurtz O., Komuro H., Fournier A. (2007). Neurotrophic effects of PACAP in the cerebellar cortex. Peptides.

[31] Ago Y., Yoneyama M., Ishihama T., Kataoka S., Kawada K., Tanaka T., Ogita K., Shintani N., Hashimoto H., Baba A. (2011). Role of endogenous pituitary adenylate cyclase-activating polypeptide in adult hippocampal neurogenesis. Neuroscience.

[32] Ogata K., Shintani N., Hayata-Takano A., Kamo T., Higashi S., Seiriki K., Momosaki H., Vaudry D., Vaudry H., Galas L. (2015). PACAP enhances axon outgrowth in cultured hippocampal neurons to a comparable extent as BDNF. PLoS ONE.

[33] Vaudry D., Hamelink C., Damadzic R., Eskay R.L., Gonzalez B., Eiden L.E. (2005). Endogenous PACAP acts as a stress response peptide to protect cerebellar neurons from ethanol or oxidative insult. Peptides.

[34] Maugeri G., D’Amico A.G., Castrogiovanni P., Saccone S., Federico C., Reibaldi M., Russo A., Bonfiglio V., Avitabile T., Longo A. (2019). PACAP through EGFR transactivation preserves human corneal endothelial integrity. J. Cell Biochem..

[35] Deguil J., Jailloux D., Page G., Fauconneau B., Houeto J., Philippe M., Muller J., Pain S. (2007). Neuroprotective effects of pituitary adenylate cyclase-activating polypeptide (PACAP) in MPP+-induced alteration of translational control in Neuro-2a neuroblastoma cells. J. Neurosci. Res..

[36] Rat D., Schmitt U., Tippmann F., Dewachter I., Theunis C., Wieczerzak E., Postina R., van Leuven F., Fahrenholz F., Kojro E. (2011). Neuropeptide pituitary adenylate cyclase-activating polypeptide (PACAP) slows down Alzheimer’s disease-like pathology in amyloid precursor protein-transgenic mice. Faseb J..

[37] Maugeri G., D’aMico A.G., Morello G., Reglodi D., Cavallaro S., D’aGata V. (2020). Differential Vulnerability of Oculomotor Versus Hypoglossal Nucleus During ALS: Involvement of PACAP. Front. Neurosci..

[38] Lee E.H., Seo S.R. (2014). Neuroprotective roles of pituitary adenylate cyclase-activating polypeptide in neurodegenerative diseases. BMB Rep..

[39] Nielsen H.S., Hannibal J., Fahrenkrug J. (1998). Expression of pituitary adenylate cyclase activating polypeptide (PACAP) in the postnatal and adult rat cerebellar cortex. Neuroreport.

[40] Di Rosa M., Castrogiovanni P., Trovato F.M., Malatino L., Ravalli S., Imbesi R. (2019). Adapted Moderate Training Exercise Decreases the Expression of Ngal in the Rat Kidney: An Immunohistochemical Study. Appl. Sci..

[41] D’amico A.G., Scuderi S., Maugeri G., Cavallaro S., Drago F., D’agata V. (2014). NAP reduces murine microvascular endothelial cells proliferation induced by hyperglycemia. J. Mol. Neurosci..

[42] D’Amico A.G., Maugeri G., Rasà D., Federico C., Saccone S., Lazzara F., Fidilio A., Drago F., Bucolo C., D’Agata V. (2019). NAP modulates hyperglycemic-inflammatory event of diabetic retina by counteracting outer blood retinal barrier damage. J. Cell Physiol..

[43] Liu P.Z., Nusslock R. (2018). Exercise-Mediated Neurogenesis in the Hippocampus via BDNF. Front. Neurosci..

[44] Yau S.-Y., Gil-Mohapel J., Christie B.R., So K.-F. (2014). Physical exercise-induced adult neurogenesis: A good strategy to prevent cognitive decline in neurodegenerative diseases?. Biomed. Res. Int..

[45] Deuel T.A., Liu J.S., Corbo J.C., Yoo S.-Y., Rorke-Adams L.B., Walsh C.A. (2006). Genetic interactions between doublecortin and doublecortin-like kinase in neuronal migration and axon outgrowth. Neuron.

[46] Zimatkin S.M., Karniushko O.A. (2016). Expression of doublecortin and neun in the developing cerebellar neurons in rat. Morfologiia.

[47] Ahlfeld J., Filser S., Schmidt F., Wefers A.K., Merk D.J., Glaß R., Herms J., Schüller U. (2017). Neurogenesis from Sox2 expressing cells in the adult cerebellar cortex. Sci. Rep..

[48] Pescatello L.S., MacDonald H.V., Lamberti L., Johnson B.T. (2015). Exercise for Hypertension: A Prescription Update Integrating Existing Recommendations with Emerging Research. Curr. Hypertens. Rep..

[49] Sosner P., Guiraud T., Gremeaux V., Arvisais D., Herpin D., Bosquet L. (2017). The ambulatory hypotensive effect of aerobic training: A reappraisal through a meta-analysis of selected moderators. Scand. J. Med. Sci. Sports.

[50] Mctiernan A., Friedenreich C.M., Katzmarzyk P.T., Powell K.E., Macko R., Buchner D., Pescatello L.S., Bloodgood B., Tennant B., Vaux-Bjerke A. (2019). Physical Activity in Cancer Prevention and Survival: A Systematic Review. Med. Sci. Sports Exerc..

[51] Rêgo M.L., Cabral D.A., Costa E.C., Fontes E.B. (2019). Physical Exercise for Individuals with Hypertension: It Is Time to Emphasize its Benefits on the Brain and Cognition. Clin. Med. Insights Cardiol..

[52] Pescatello L.S., Parducci P., Livingston J., Taylor B.A. (2019). A Systematically Assembled Signature of Genes to be Deep-Sequenced for Their Associations with the Blood Pressure Response to Exercise. Genes.

[53] Krüger K., Mooren F.C., Pilat C. (2016). The Immunomodulatory Effects of Physical Activity. Curr. Pharm. Des..

[54] Pedersen B.K. (2017). Anti-inflammatory effects of exercise: Role in diabetes and cardiovascular disease. Eur. J. Clin. Investig..

[55] Di Liegro C.M., Schiera G., Proia P., Di Liegro I. (2019). Physical Activity and Brain Health. Genes.

[56] Ben Ezzdine L., Dhahbi W., Dergaa I., Ceylan H., Guelmami N., Ben Saad H., Chamari K., Stefanica V., El Omri A. (2025). Physical activity and neuroplasticity in neurodegenerative disorders: A comprehensive review of exercise interventions, cognitive training, and AI applications. Front. Neurosci..

[57] Farmand S., Du Preez A., Kim C., de Lucia C., Ruepp M.-D., Stubbs B., Thuret S. (2025). Cognition on the move: Examining the role of physical exercise and neurogenesis in counteracting cognitive aging. Ageing Res. Rev..

[58] Couillard-Despres S., Winner B., Schaubeck S., Aigner R., Vroemen M., Weidner N., Bogdahn U., Winkler J., Kuhn H., Aigner L. (2005). Doublecortin expression levels in adult brain reflect neurogenesis. Eur. J. Neurosci..

[59] Takács J., Zaninetti R., Víg J., Vastagh C., Hámori J. (2008). Postnatal expression of Doublecortin (Dcx) in the developing cerebellar cortex of mouse. Acta Biol. Hung..

[60] Gleeson J.G., Lin P.T., A Flanagan L., A Walsh C. (1999). Doublecortin is a microtubule-associated protein and is expressed widely by migrating neurons. Neuron.

[61] Boseret G., Ball G.F., Balthazart J. (2007). The microtubule-associated protein doublecortin is broadly expressed in the telencephalon of adult canaries. J. Chem. Neuroanat..

[62] Waschek J.A. (2013). VIP and PACAP: Neuropeptide modulators of CNS inflammation, injury, and repair. Br. J. Pharmacol..

[63] Mansouri S., Agartz I., Ögren S.-O., Patrone C., Lundberg M. (2017). PACAP Protects Adult Neural Stem Cells from the Neurotoxic Effect of Ketamine Associated with Decreased Apoptosis, ER Stress and mTOR Pathway Activation. PLoS ONE.

[64] Stumm R., Kolodziej A., Prinz V., Endres M., Wu D., Höllt V. (2007). Pituitary adenylate cyclase-activating polypeptide is up-regulated in cortical pyramidal cells after focal ischemia and protects neurons from mild hypoxic/ischemic damage. J. Neurochem..

[65] Riek-Burchardt M., Kolodziej A., Henrich-Noack P., Reymann K.G., Höllt V., Stumm R. (2010). Differential regulation of CXCL12 and PACAP mRNA expression after focal and global ischemia. Neuropharmacology.

[66] Lin C.-H., Chiu L., Lee H.-T., Chiang C.-W., Liu S.-P., Hsu Y.-H., Lin S.-Z., Hsu C.Y., Hsieh C.-H., Shyu W.-C. (2015). PACAP38/PAC1 signaling induces bone marrow-derived cells homing to ischemic brain. Stem Cells.

[67] Skoglösa Y., Lewén A., Takei N., Hillered L., Lindholm D. (1999). Regulation of pituitary adenylate cyclase activating polypeptide and its receptor type 1 after traumatic brain injury: Comparison with brain-derived neurotrophic factor and the induction of neuronal cell death. Neuroscience.

[68] Ohta S., Gregg C., Weiss S. (2006). Pituitary adenylate cyclase-activating polypeptide regulates forebrain neural stem cells and neurogenesis in vitro and in vivo. J. Neurosci. Res..

[69] Waschek J.A., Casillas R.A., Nguyen T.B., DiCicco-Bloom E.M., Carpenter E.M., Rodriguez W.I. (1998). Neural tube expression of pituitary adenylate cyclase-activating peptide (PACAP) and receptor: Potential role in patterning and neurogenesis. Proc. Natl. Acad. Sci. USA.

[70] Perényi H., Szegeczki V., Horváth G., Hinnah B., Tamás A., Radák Z., Ábrahám D., Zákány R., Reglodi D., Juhász T. (2020). Physical Activity Protects the Pathological Alterations of Alzheimer’s Disease Kidneys via the Activation of PACAP and BMP Signaling Pathways. Front. Cell Neurosci..

