# Supplementary material for: Moderate Exercise Stimulates PACAP-Mediated Neurogenesis in Rat Dentate Gyrus and Cerebellar Cortex
Source: J Funct Morphol Kinesiol. 2026 Jan 15;11(1):37. doi: 10.3390/jfmk11010037 (PMC12821592; doi:10.3390/jfmk11010037)
Supplement: Supplementary file 1 [file jfmk-11-00037-s001.zip › jfmk-4056520-supplementary.pdf]

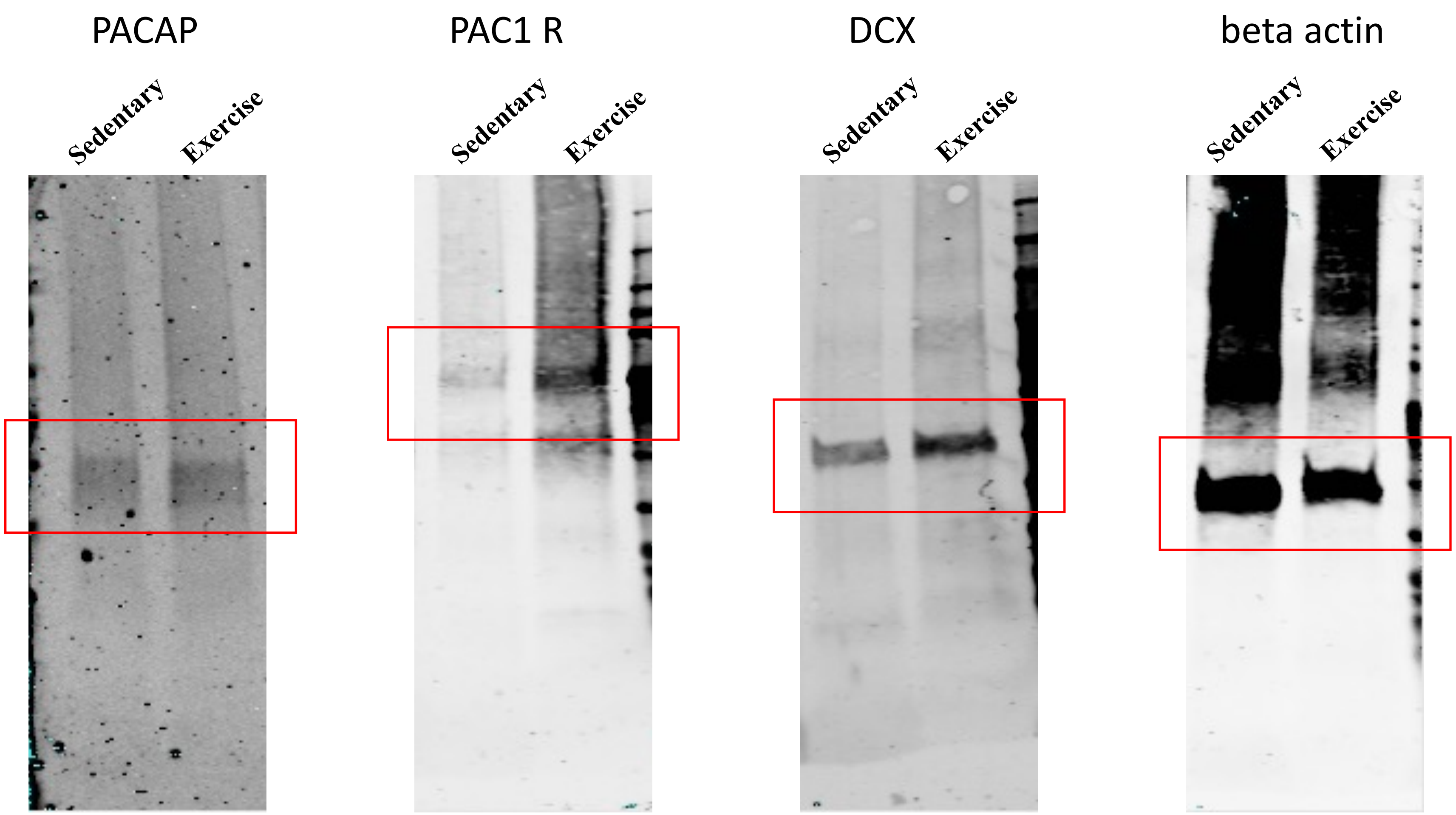

Supplementary Figure S1. – Original Western Blot. Evaluation of PACAP, PAC1R, DCX and β-actin in DG of sedentary and exercise group.

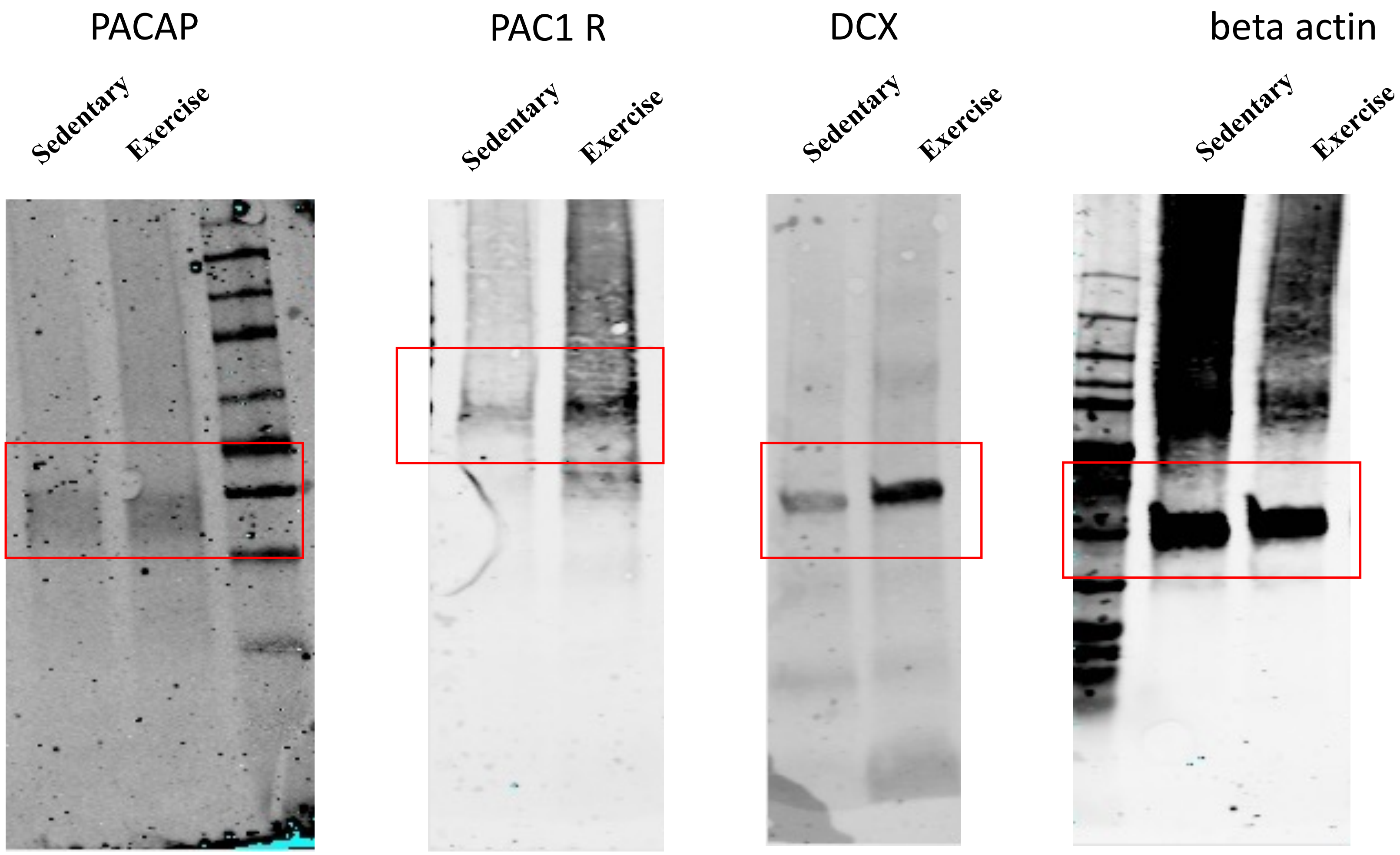

Supplementary Figure S2. – Original Western Blot. Evaluation of PACAP, PAC1R, DCX and β-actin in cerebellar cortex of sedentary and exercise group.
